# Multi-omics data elucidate parasite-host-microbiota interactions and resistance to *Haemonchus contortus* in sheep

**DOI:** 10.1186/s13071-024-06205-9

**Published:** 2024-03-01

**Authors:** Simone Cristina Méo Niciura, Tainã Figueiredo Cardoso, Adriana Mercia Guaratini Ibelli, Cintia Hiromi Okino, Bruno Gabriel Andrade, Magda Vieira Benavides, Ana Carolina de Souza Chagas, Sergio Novita Esteves, Alessandro Pelegrine Minho, Luciana Correia de Almeida Regitano, Cedric Gondro

**Affiliations:** 1 Embrapa Pecuária Sudeste, São Carlos, SP Brazil; 2 Munster Technological University, Cork, Ireland; 3 Embrapa Pecuária Sul, Bagé, RS Brazil; 4 Michigan State University, East Lansing, MI USA

**Keywords:** GWAS, RNA-seq, 16S rDNA-sequencing, eQTL, Parasite resistance, Barber’s pole worm

## Abstract

**Background:**

The integration of molecular data from hosts, parasites, and microbiota can enhance our understanding of the complex biological interactions underlying the resistance of hosts to parasites. *Haemonchus contortus*, the predominant sheep gastrointestinal parasite species in the tropics, causes significant production and economic losses, which are further compounded by the diminishing efficiency of chemical control owing to anthelmintic resistance. Knowledge of how the host responds to infection and how the parasite, in combination with microbiota, modulates host immunity can guide selection decisions to breed animals with improved parasite resistance. This understanding will help refine management practices and advance the development of new therapeutics for long-term helminth control.

**Methods:**

Eggs per gram (EPG) of feces were obtained from Morada Nova sheep subjected to two artificial infections with *H. contortus* and used as a proxy to select animals with high resistance or susceptibility for transcriptome sequencing (RNA-seq) of the abomasum and 50 K single-nucleotide genotyping. Additionally, RNA-seq data for *H. contortus* were generated, and amplicon sequence variants (ASV) were obtained using polymerase chain reaction amplification and sequencing of bacterial and archaeal 16S ribosomal RNA genes from sheep feces and rumen content.

**Results:**

The heritability estimate for EPG was 0.12. *GAST*, *GNLY*, *IL13*, *MGRN1*, *FGF14*, and *RORC* genes and transcripts were differentially expressed between resistant and susceptible animals. A genome-wide association study identified regions on chromosomes 2 and 11 that harbor candidate genes for resistance, immune response, body weight, and adaptation. *Trans*-expression quantitative trait loci were found between significant variants and differentially expressed transcripts. Functional co-expression modules based on sheep genes and ASVs correlated with resistance to *H. contortus*, showing enrichment in pathways of response to bacteria, immune and inflammatory responses, and hub features of the *Christensenellaceae*, *Bacteroides*, and *Methanobrevibacter* genera; *Prevotellaceae* family; and *Verrucomicrobiota* phylum. In *H. contortus*, some mitochondrial, collagen-, and cuticle-related genes were expressed only in parasites isolated from susceptible sheep.

**Conclusions:**

The present study identified chromosome regions, genes, transcripts, and pathways involved in the elaborate interactions between the sheep host, its gastrointestinal microbiota, and the *H. contortus* parasite. These findings will assist in the development of animal selection strategies for parasite resistance and interdisciplinary approaches to control *H. contortus* infection in sheep.

**Graphical Abstract:**

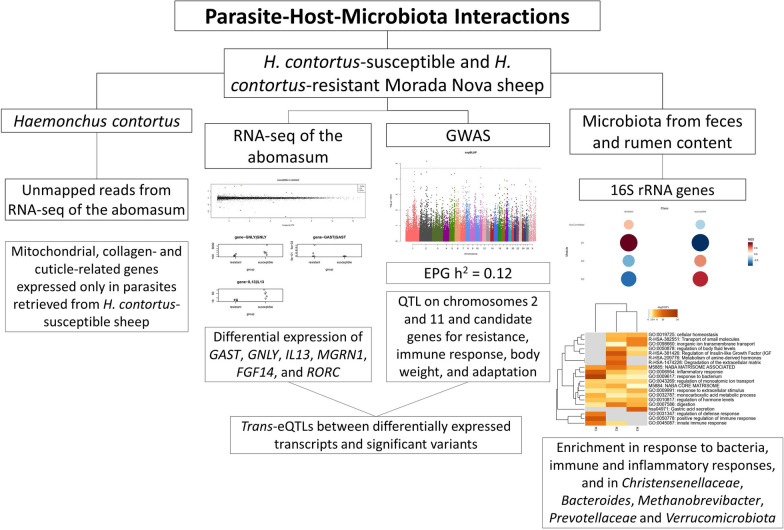

**Supplementary Information:**

The online version contains supplementary material available at 10.1186/s13071-024-06205-9.

## Background

Helminths cause significant economic and production losses in small ruminant livestock systems [1]. *Haemonchus contortus*, the barber’s pole worm, is the most prevalent pathogenic nematode affecting small ruminants in tropical regions [2]. In contrast, chemical treatments, which are the main approaches currently used for its control, are becoming less effective owing to the development of anthelmintic resistance [3].

Therefore, there is a need to develop new alternative control strategies to reduce the parasite burden on production systems. One interesting animal-centric control strategy is to use sheep breeds that are naturally more resistant to gastrointestinal nematodes or, alternatively, to select more resistant individuals within breeds [4]. Some indigenous breeds have adapted to survive with low maintenance requirements and high parasitic challenges [5]. An example of such a breed is the Morada Nova, a native Brazilian hair sheep breed that has high gastrointestinal nematode resistance compared to other tropical-adapted and high-productive sheep breeds [6]. Better molecular characterization of Morada Nova can support the use of this indigenous breed in production systems as a long-term helminth control strategy [7] and advance our understanding of the mechanisms of immune responses and parasite resistance in other sheep breeds.

Knowledge of pathology of haemonchosis is essential for establishing new therapeutics and control strategies. This can be assisted by omics technologies, which can be used to increase our understanding of helminth and host responses [8]. Multi-omics approaches can aggregate various layers of information to elucidate complex host-parasite interactions [9], particularly those that are regulated by several genes with minor effects and are influenced by diverse environmental conditions. Genomics can reveal the genetic mechanisms underlying parasite resistance in hosts, whereas transcriptomics can identify the mechanisms through which hosts respond to infections, or those used by parasites to establish infection and modulate the host immune system. Integrating both host and parasite data quantified through genomic and transcriptomic layers, such as those used in expression quantitative trait loci (eQTL) studies [10], can provide a more in-depth knowledge of interactions.

Another factor in the parasite-host molecular scenario is the host microbiota, which has received increasing attention in recent years owing to the effect of the microbiome on resistance to gastrointestinal nematodes [11], *H. contortus* burden [12], and *Teladorsagia circumcincta* female fertility [13] in sheep. Symbiosis between microorganisms and their hosts, particularly ruminants, is crucial for providing nutrients, carbohydrates, and proteins [14]. However, gastrointestinal nematode infections alter the typical host microbiota, resulting in microenvironmental dysbiosis [15, 16]. *H. contortus* infections change the bacterial genera in the abomasum and rumen [15], and *T. circumcincta* increases the bacterial population involved in pro-inflammatory processes, which may contribute to the immunopathology of helminth disease [16]. Furthermore, the microbiota may influence parasite and host gene expression, as they can interact with host/parasite cells and affect gene regulation, switching certain cellular processes on or off.

The aim of this study was to improve our understanding of the parasite-host-microbiota interactions that lead to increased resistance to *H. contortus* in Morada Nova sheep using an integrated multi-omics approach with transcriptome sequencing (RNA-seq) data from sheep abomasum and *H. contortus*, 50 K sheep genotypes, and 16S ribosomal RNA (rRNA) gene sequencing of the microbiota from sheep feces and rumen content.

## Methods

### Sample collection and phenotypes

#### Sheep

The Morada Nova sheep and the phenotypes used in this study have been previously described [17]. Briefly, 274 lambs (136 males and 138 females), the progeny of four sires and 156 dams from the Embrapa Pecuária Sudeste flock, were born in two breeding seasons (March to May 2017 and 2018). All lambs were raised under the same environmental conditions.

### Parasitological examination

For egg per gram (EPG) counts, 2 *g* of feces collected from the rectum were mixed with 28 mL saturated sodium chloride solution and evaluated in a McMaster chamber, then the total number of counted gastrointestinal strongyles eggs was multiplied by 50 [18].

Fecal cultures [19] performed at the end of each artificial infection revealed 100% *Haemonchus*.

### Artificial infection and phenotyping

Lambs (3 months old) were treated with monepantel (2.5 mg/kg; Zolvix^®^) to eliminate natural infection (96.4% *Haemonchus*, 2.1% *Cooperia*, and 1.5% *Trichostrongylus*) with gastrointestinal nematodes. After 2 weeks, the animals were experimentally infected with 4000 third-stage larvae (L_3_) of the Echevarria *H. contortus* isolate [20]. This isolate, retrieved in Brazil in 1991, exhibited anthelmintic susceptibility to levamisole (100%), ivermectin (100%), and albendazole (98.9%) [20], and was obtained before the launch of monepantel on the market.

Phenotyping of individuals was based on EPG obtained on day 0 (day of infection) and in four collections at 7-day intervals after *H. contortus* prepatent period [21], from days 21–42 (days 21, 28, 35, and 42 after infection). On day 42 of the first artificial infection, the lambs were treated again with monepantel and, after 15 days, were subjected to a new parasitic infection with the same *H. contortus* isolate, following the same sampling protocol. On day 42 of the second parasitic challenge, the EPG mean was calculated from the ten collection dates, and the animals were ordered based on the mean EPG and ranked as resistant (EPG mean ranging 10–1765) or susceptible (EPG mean ranging 3923–24,112) to *H. contortus*. Notably, the natural infection, comprising *H. contortus* with historical multidrug resistance [22], was not totally eliminated after the first and second monepantel treatments, resulting in EPG counts on day 0 in both artificial challenges (Table 1). Therefore, the day of infection (day 0) was included in the calculation of the EPG mean.

**Table 1:** Mean and standard deviation of eggs per gram (EPG) during *Haemonchus contortus* challenges in Morada Nova lambs

ID	Sex	Sire	Dam	Days at the first challenge					Days at the second challenge					Mean	SD	Rank
				0	21	28	35	42	0	21	28	35	42			
819	F	3	210	0	0	0	0	0	0	0	0	0	100	10	32	R
783	F	1	166	0	0	0	50	0	0	0	0	0	100	15	34	R
886	F	3	401	0	0	0	50	50	0	50	0	0	0	15	24	R
803	F	3	370	0	0	0	0	0	0	0	0	50	100	15	34	R
795	F	4	230	0	50	0	0	100	0	0	50			25	38	R
836^a^	F	1	289	0	50	0	0	150	100	0	100	150	200	75	75	R
897^a^	F	2	489	0	100	100	50	0	0	200	350	50	0	85	113	R
893	F	2	389	0	100	0	1000	350	0	0	50	0	0	150	318	R
883^a^	F	4	532	50	0	200	100	100	50	100	250	450	600	190	194	R
949	F	4	439	0	0	0	0	0	0	50	0	100	2100	225	660	R
818^a^	M	3	210	0	0	0	100	550	0	50	850	700	0	225	337	R
805	F	1	283	0	100	50	900	400	0	0	50	700	500	270	333	R
732^a^	F	1	382	0	0	250	1850	1450	50	0	0	450	50	410	676	R
762	M	4	7	150	0	800	150	2850	0	50	250	550	650	545	858	R
891	M	1	249	0	100	350	2950	2600	500	400	100	300	650	795	1065	R
988	M	3	333	200	400	1050	1950	2450	350	50	150	1500	500	860	841	R
789	M	4	177	0	100	150	600	1300	50	350	2100	2700	2200	955	1034	R
917	M	4	254	200	100	900	3100	1800	550	1000	1950	800	500	1090	932	R
797	M	3	403	0	300	350	700	3000	2000	1700	850	600	2100	1160	979	R
918	M	4	508	0	1750	4700	2750	100	350	1550	800	900	1400	1430	1422	R
894	M	2	389	150	400	3250	5600	4200	400	500	1600	700	850	1765	1908	R
906	F	2	503	100	700	5350	9400	11,300	600	3200	2250	1500	1050	3545	3923	S
944	F	2	542	150	2950	7850	17,300	8850	400	700	250	200	500	3915	5732	S
985	F	3	368	0	550	7950	21,750	26,450	1600	0	0	100	1200	5960	9916	S
921	M	2	122	50	8000	12,500	12,500	7400	1400	6350	1200	5800	6100	6130	4347	S
898	M	2	431	50	14,800	17,100	12,000	16,650	650	1200	1000	800	400	6465	7589	S
740	F	3	349	50	1450	9100	6200	4000	0	3350	15,700	13,200	11,950	6500	5692	S
1038	F	3	252	50	7500	12,100	18,300	19,350	400	500	700	3550	3800	6625	7461	S
907	M	3	415	400	10,450	13,000	12,100	3850	650	4100	2550	900	18,400	6640	6340	S
756	F	3	527	0	15,000	14,550	100	100	0	2650	14,000	15,600	13,000	7500	7376	S
852^a^	M	1	391	100	3500	19,000	20,000	22,000	0	3000	1100	8700	2750	8015	8871	S
739	M	3	349	50	3700	26,250	10,550	6200	150	3350	9000	9700	14,200	8315	7811	S
1053^a^	F	1	299	0	5400	6550	18,750	33,550	350	500	4000	4950	9500	8355	10,447	S
718	M	1	252	0	3150	12,700	14,950	10,600	50	4700	12,450	6500	20,700	8580	6821	S
923^a^	F	1	223	0	8800	11,700	15,850	10,200	600	5150	13,600	8000	12,300	8620	5309	S
742	M	1	401	0	50	12,100	10,550	25,100	0	1450	13,050	8350	20,000	9065	8870	S
727	M	1	184	50	100	7350	7350	9200	0	4100	10,500	25,000	37,200	10,085	12,072	S
1013^a^	M	3	509	300	6000	21,200	20,500	35,000	9150	1500	3300	9800	7400	11,415	10,931	S
858	F	4	387	50	10,450	25,000	15,850	38,450	0	5450	8700	15,000	9000	12,795	11,729	S
863	F	4	282		7300	16,000	36,500	56,100	0	3000	3550	12,200	12,500	16,350	18,404	S
781^a^	M	4	339	100	150	20,350	21,900	8900	0	11,000	36,500	45,950	31,100	17,595	16,361	S
1049	F	4	486	0	1400	12,400	34,450	44,200	5300	14,450	40,000	14,450		18,517	16,793	S
1052	M	4	296	300	16,000	28,100	46,150	49,300	9450	12,000	16,100	30,900	25,800	23,410	15,772	S
1048	M	4	382	50	16,250	22,600	39,500	42,500	7350	22,500	27,500	81,950		28,911	24,112	S

^a^ Animals selected for RNA-seq. *ID* animal identification, *SD* standard deviation, *M* male, *F* female, *R* resistant, *S* susceptible

All four sires and 79 of the 156 dams were phenotypically evaluated in two parasite challenges, following the same protocol used for the lambs, between September 2018 and January 2019.

### Statistical analyses for heritability estimation

Considering the Morada Nova lamb-sire-dam trios, EPG phenotypic information for four sires, 79 dams, and 147 lambs, totaling 230 individuals, were available. These animals were used to build the additive genetic relationship matrix (A matrix) using the AGHmatrix [23] and pedigreemm [24] R (version 4.2.1 [25]) packages, and heritability (h^2^) was estimated using the NAM package [26].

### Selection of lambs for the genome-wide association study (GWAS)

A total of 44 lambs from the top- and bottom-ranked animals (Table 1) were split into two groups with high and low EPG means, with a 21.5 × variation in EPG means between the groups. Lambs were also selected to balance the sex distribution between the groups and, as much as possible, the number of offspring per sire. Blood samples from each of the four sires and 44 lambs were collected through the jugular vein and subjected to DNA extraction [27]. All 48 DNA samples were genotyped using an Illumina 50 K single-nucleotide polymorphism (SNP) array.

### Selection of lambs for transcriptome and metabarcoding sequencing

Five resistant and five susceptible animals were selected for transcriptome sequencing (RNA-seq) and metabarcoding (16S rRNA) sequencing. As expected, an unequal distribution of genders occurred [28] with a larger number of females in the resistant group. To better characterize the local host response in the early phase of infection [29], these ten lambs were dewormed with monepantel, allocated to cemented stalls, subjected to a third challenge with 4000 L_3_ from the same *H. contortus* isolate, and euthanized 7 days later. The abomasum was retrieved, and fragments of the fundic region were collected, immediately frozen in liquid nitrogen without any additional processing, and stored at – 80 ℃ for RNA extraction. Furthermore, 10 *g* of feces from the rectal ampulla was collected 2 weeks before slaughter, and 50 mL of rumen content of the slaughtered sheep was stored in liquid nitrogen for microbiota investigation.

### RNA-seq

Total RNA was extracted from fragments of the fundic region of the abomasum using QIAzol Lysis Reagent (Qiagen, Germany) and TissueRuptor (Qiagen, Germany), and purified in a silica column with an RNeasy Mini kit (Qiagen, Germany). RNA concentration (ng/µL) and purity (A260/A280 ratio) were estimated in Qubit using an HS RNA kit (Thermo Fisher Scientific, USA) and in NanoDrop 2000 spectrophotometer (Thermo Fisher Scientific, USA), respectively. RNA samples were submitted to RNA-seq after quality confirmation through RNA Integrity Number ≥ 7.5 in Agilent 2100 Bioanalyzer (Agilent Technologies, USA). RNA-seq libraries were prepared using the TruSeq Stranded mRNA kit (Illumina, USA) and sequenced using an Illumina HiSeq2500 system. For each sample, approximately 40 million reads in a 100 bp paired-end (PE) protocol were sequenced.

After sequencing, FASTQ files were visualized using FastQC (version 0.11.7 [30]) to assess RNA-seq quality. Trimmomatic (version 0.39 [31]) was used to remove adapters, the first ten initial bases and 2–3 final bases, reads of < 50 bases, and reads with a phred score of < 15 in a 4-bp window.

Initially, three reference sheep genomes were compared: Oar_rambouillet_v1.0 (GCA_002742125.1), Oar_v3.1 Texel (GCA_000298735.1), and ARS-UI_Ramb_v2.0 (GCF_016772045.1) (data not shown). As ARS-UI_Ramb_v2.0 (version 104) showed higher alignment rates and number of transcripts compared to the others, and this genome assembly was selected as the reference for subsequent analysis. HISAT2 (version 2.1.0 [32]) was used to index the sheep reference genome and align the trimmed paired reads. The resultant SAM files were converted to BAM files using SAMtools (version 1.11 [33]), and statistics were generated using MultiQC (version 1.7 [34]). The aligned and sorted BAM files were assembled using StringTie (version 2.1.3 [35]). Gene and transcript read counts were extracted using the Python (version 3.6.6 [36]) prepDE.py3 script in the StringTie pipeline.

For differential gene expression analysis between susceptible and resistant animals, an initial filtering step in R software was used to remove lowly expressed genes with a count sum of < 50, and overexpressed genes with a mean count of > 100,000. The gene count matrix was analyzed using DESeq2 [37], with a Wald test adjusted *p*-value (*q*-value) of < 0.1, and using edgeR [38], with a false discovery rate (FDR) of < 0.05. Differential transcript expression was analyzed in ballgown [39] (*q*-value < 0.1) and DESeq2 (*q*-value < 0.05). In all analyses, both | fold change (FC) |> 1.5 and adjusted *p*-values were regarded as thresholds. Regarding *p*-values, in the absence of results at < 0.05, an adjusted *p*-value of < 0.1 was used. The differential expression of the overexpressed genes was investigated separately using the t-test, DESeq2, and edgeR. The biological roles of the differentially expressed and overexpressed genes were investigated using GeneCards (version 5.15 [40]).

### Variant calling and annotation of RNA-seq data

For variant calling from the abomasum RNA-seq data [41], trimmed PE reads were mapped to the indexed sheep reference genome using the 2-pass mode of STAR (version 2.7.3a [42]). The STAR aligner was used because of its higher tolerance for accepting mismatches and soft clipping compared with the HISAT2 [43]. SAMtools was used to index the BAM files, and Picard (version 2.25.0 [44]) was used to add read groups, mark duplicates, and create a sequence dictionary. GATK (version 4.2.0.0 [45]) with SplitNCigar, BaseRecalibrator including known sheep variants [46], ApplyBQSR, and HaplotypeCaller, was used to call the variants. GATK GenomicsDBImport was used to combine files from different animals, and GenotypeGVCFs was used for joint genotyping. GATK SelectVariants was used to select the SNPs, which were then filtered with VariantFiltration with –cluster-window-size 35, –cluster-size 3, QD < 2.0, FS > 30.0, SOR > 3.0, MQ < 40.0, MQRankSum < − 12.5, and ReadPosRankSum < − 8.0. BCFtools (version 1.9.64 [33]) was used to retrieve hard-filtered variants and remove poor-quality calls per variant (GQ < 20, depth < 3 reads, no calls, MAF < 0.05, missing genotypes > 10%, and multiallelic sites). A total of 51,434 SNPs were identified.

Subsequently, a database for the ARS-UI_Ramb_v2.0 sheep reference genome was created and the variants were annotated using SnpEff (version 5.1 [47]). SnpSift [48] was used to detect high-impact variants, and the caseControl function was used to select homozygous variants between resistant and susceptible sheep (termed RNA-seq genotypes).

### Genome-wide association study

DNA from the four sires and 44 extreme phenotype lambs, including the ten lambs used in the RNA-seq study, were extracted through saline precipitation and stored at – 20 ℃. DNA integrity in 1% agarose gel electrophoresis, concentration (ng/µL) and purity (260/280 absorbance ratio ranging 1.8–2.0) in NanoDrop were assessed. The DNA samples were genotyped using an Illumina OvineSNP50v3 chip. Genotyping data were subjected to quality control with snpQC [49], and SNPs with GCScore < 0.5, MAF < 0.05, HWE < 10e-16, call rate < 0.90, and samples with call rate < 0.75 were assigned as missing values. No samples with > 10% missing values were detected. A total of 45,070 SNPs and all 48 samples were included in the following steps. The genotype frequency of SNPs with missing data was imputed using the mean frequency of the other samples. Paternity was verified using a matrix of opposing homozygotes constructed using R [50].

These data were previously used for a case–control GWAS [51] based on the Oar_v3.1 Texel reference genome. In the present study, data from the 44 lambs were reanalyzed using the same reference genome as that used in the RNA-seq analysis. A custom script was used to lift the 50 K genotypes to the ARS-UI_Ramb_v2.0 assembly by mapping the probe sequence to the reference and then re-aligning the nucleotides to the reference strand. The EPG mean normalized by cube-root transformation was used as a phenotype in an snpBLUP GWAS to identify significant SNPs at FDR < 0.05, and h^2^ for EPG was estimated from the genomic data with the NAM package in R. Only the genotypes from the arrays were used for the GWAS, as no significant results (data not shown) were obtained using the RNA-seq genotypes from the ten lambs.

Genes located 1 Mbp upstream and downstream of each significant SNP from the GWAS analysis were retrieved from the National Center for Biotechnology Information (NCBI) Genome Data Viewer [52], and gene function was investigated using GeneCards and a literature review to identify candidate genes for resistance to *H. contortus*.

### eQTL analysis

The R MatrixEQTL package [53] was used for eQTL analysis using all SNP data (45,070 from the array and 51,434 from RNA-seq genotypes) and the RNA-seq of the ten most extreme animals. Gene and transcript expressions were RNA-seq read counts normalized by DESeq2 variance-stabilizing transformation. Sex and sire were included as covariates. *Cis*-eQTL were defined as SNPs located up to 1 Mb from the regulated gene, whereas *trans*-eQTL considered distances of > 1 Mb. From all significant *cis*- and *trans*-eQTL (FDR < 0.05) retrieved, only those comprising significant SNPs from GWAS or RNA-seq genotypes and differentially expressed data are reported here.

### Analysis of unmapped RNA-seq reads

The RNA-seq study was originally designed to assess gene expression in sheep hosts, but as *H. contortus* L_3_ penetrates the mucosal glands of the abomasum, primarily in the fundic region, to molt into L_4_ [54], the presence of genetic material from the *H. contortus* parasite in the RNA-seq data was also investigated using reads unmapped to the sheep reference genome. The trimmed PE reads were aligned to the *H. contortus* reference genome (*Haemonchus_contortus*.PRJEB506.WBPS17.genomic.fa) and assembled with an annotation file (*Haemonchus_contortus*.PRJEB506.WBPS17.annotations.gff3) retrieved from the WormBase ParaSite database (version 17 [55]).

Because of the small amount of data and the resultant low read counts, no additional filtering was performed, and differential expression analysis was implemented through DESeq2, edgeR, ballgown, and t-tests using the same parameters previously described for sheep.

### Microbiota from sheep feces and rumen content

DNA from the feces and rumen content of the ten lambs selected for RNA-seq was extracted using the Quick-DNA^™^ Fecal/Soil Microbe Miniprep Kit (Zymo, USA) following the manufacturer’s instructions. Polymerase chain reaction (PCR) amplification of the bacterial and archaeal 16S rRNA genes was performed using a previously described primer set [56]. Libraries were sequenced on an Illumina MiSeq (2 × 250 bp) using an Illumina V3 sequencing kit (Illumina, USA). Raw reads were quality checked, filtered for quality (> Q25), and trimmed at positions 220 (forward) and 175 (reverse), based on aggregated quality plots generated using QIIME 2 [57]. The remaining data were submitted to DADA2 [58] to resolve the sequences into amplicon sequence variants (ASV) and exclude chimeric sequences. Bacterial and archaeal sequences were classified using the SILVA database (version 132 [59]). The conditional quantile regression (ConQuR) approach [60] was used to remove the batch effects caused by multiple sequencing runs and sex. Only the ASVs present in at least five lambs were considered. A total of 526 and 607 bacterial ASVs and 22 and 28 archaeal ASVs from the feces and rumen, respectively, were considered for downstream analyses.

### Multi-omics integration

The final step of the analysis was the integration of fecal and ruminal microbiota abundance identified in the 16S rRNA data with the expression identified using RNA-seq. Co-expression network analysis of ASVs, host genes/transcripts, and *H. contortus* gene/transcript expression was performed using the R CEMiTool package [61]. CEMiTool uses an unsupervised filtering method based on the inverse gamma distribution for each gene selection used in the analyses and applies a soft-thresholding power β to determine a similarity criterion between pairs of features [61]. The CEMiTool parameters were set to apply_vst = TRUE, cor_method = “spearman”, network_type = “signed”, and tom_type = “signed” to construct co-expression networks. The minimum module size was set to 50 and 100 for gene and transcript expression levels, respectively. Genes within significantly associated modules were then subjected to Metascape [62] for biological pathway and gene ontology enrichment analyses, with the input species set to *Homo sapiens*.

## Results and discussion

### RNA-seq

The sheep abomasum RNA-seq reads ranged 31.1–41.3 M per sample, with 97.9–98.5% of the reads mapped to the sheep ARS-UI_Ramb_v2.0 reference genome (64.5–70% of proper pairs). For the differential gene expression analysis, 9099 genes with zero counts and 5891 genes with count sums < 50 were removed from the total of 33,800 genes.

Fourteen overexpressed genes (Table 2) were removed and analyzed separately; and no significant differential expression was detected between resistant and susceptible animals. Among these overexpressed genes, *PIGR*, *COX1*, and *LGALS15* have been reported in the sheep abomasum in a transcriptome study comparing several tissues [63], and *PIGR* was the most abundant transcript found in the cattle abomasum [64]. These overexpressed genes are involved in gastrointestinal biological processes and infection responses. Pepsin is the principal acid protease of the stomach [65]; lysozymes are involved in bacterial cell wall cleavage [66]; *COX1* produces cytoprotective prostaglandins for the stomach [67]; *LGALS15* (also known as *ovgal11*), associated with immune/inflammatory responses and protection against infection [68], and intelectin-2, associated with the gastrointestinal mucus [69], are present in the abomasum of sheep infected with *H. contortus*; immunoglobulin lambda expression is detected in gastric cancer [70]; and *ND4* mutations are considered biomarkers of preneoplastic lesions of the gastrointestinal tract [71].

**Table 2:** Overexpressed genes in RNA-seq of the abomasum of Morada Nova sheep

Gene ID	Gene name
*LOC101105864*	Pepsin A
*LOC443320*	Lysozyme C-1-like
*LOC443322*	Lysozyme 3a precursor
*COX1*	Cytochrome c oxidase subunit I
*PIGR*	Polymeric immunoglobulin receptor
*COX3*	Cytochrome c oxidase subunit III
*LGALS15*	Lectin, galactoside-binding, soluble, 15
*LOC114108841*	Immunoglobulin lambda variable 2–14
*LOC101122151*	Intelectin 2
*ATP6*	ATP synthase F0 subunit 6
*ND4*	NADH dehydrogenase subunit 4
*LOC121818276*	NADH-ubiquinone oxidoreductase chain 1-like
*LOC101107475*	Ig alpha-1 chain C region-like
*COX2*	Cytochrome c oxidase subunit II

Of the remaining 18,796 filtered genes (55.6%), 11 differentially expressed genes (DEG) (Table 3) were detected between *H. contortus*-susceptible and *H. contortus*-resistant animals using edgeR (Additional file 1: Table S1 and Additional file 3:Fig. S1). Four genes (*CCDC85B*, *LOC121819799*, *GAST*, and *LOC101121371*) were upregulated in *H. contortus*-resistant sheep, whereas seven genes (*LOC114116426*, *LOC101116991*, *LOC101104728*, *LOC101107420*, *GNLY*, *GRP*, and *IL13*) were upregulated in susceptible sheep. Using DESeq2 (Additional file 1: Table S2), only two DEGs were upregulated in susceptible sheep (*LOC101116991* and *LOC114116426*; both were DEGs shown in the edgeR results) (Table 3). The role of *IL13* in the resistance to *H. contortus* is through smooth muscle hypercontraction and increased mucus production, resulting in the detachment of parasites from the gut wall [72]. *IL13* upregulation in the susceptible Morada Nova is in accordance with a previous study using infected young sheep of a native breed [73]. However, *IL13* has also been shown to be upregulated in adult resistant sheep [72]. In addition to animal age, infection duration should be considered, as genes related to the inflammatory response, T lymphocyte attraction, and leukocyte binding are upregulated in the abomasal lymph nodes of resistant animals at the beginning of *T. circumcincta* infection but downregulated at later stages, indicating a delayed Th2 response in susceptible animals [29]. Associations between the *GAST* gene and the response to *H. contortus*[74] and between the *GNLY* gene and the response to *Trichostrongylus colubriformis* [75] have been previously reported.

**Table 3:** Differentially expressed genes in the abomasum between *H. contortus*-susceptible and resistant Morada Nova sheep using edgeR and DESeq2

Gene ID	Log FC	FDR/*q*-value	Gene name	Upregulated
edgeR				
* CCDC85B*	− 8.95	0.0011	Coiled-coil domain containing 85B	Resistant
* LOC121819799*	− 6.67	0.0060	ncRNA	Resistant
* GAST*	− 13.43	0.0158	Gastrin	Resistant
* LOC101121371*	− 3.15	0.0257	60S ribosomal protein L37a	Resistant
* LOC114116426*^a^	7.33	0.0004	Protein C1orf43 homolog (pseudogene)	Susceptible
* LOC101116991*^a^	2.17	0.0060	Ribonuclease K6-like	Susceptible
* LOC101104728*	8.36	0.0060	Antimicrobial peptide NK-lysin	Susceptible
* LOC101107420*	3.98	0.0074	Phospholipase A2, membrane associated	Susceptible
* GNLY*	3.45	0.0141	Granulysin	Susceptible
* GRP*	9.09	0.0232	Gastrin releasing peptide	Susceptible
* IL13*	2.10	0.0474	Interleukin 13	Susceptible
DESeq2				
* LOC101116991*^a^	2.17	0.060	Ribonuclease K6-like	Susceptible
* LOC114116426*^a^	6.81	0.055	Protein C1orf43 homolog (pseudogene)	Susceptible

^a^ Detected in both edgeR and DESeq2 analyses

A subset of ten genes (expressed in at least two samples and count sum of > 50 reads) was observed to be exclusively expressed in susceptible or resistant animals. Of these, six genes (*CCDC85B*, *LOC121819799*, *GAST*, *ABCC11*, *LOC101106468*, and *LOC121818066*) were expressed only in resistant sheep and four genes (*LOC101104728*, *GNGT1*, *LOC114116426*, and *GRP*) were expressed only in susceptible sheep (Table 4). Six of these genes (*CCDC85B*, *LOC121819799*, *GAST*, *LOC101104728*, *LOC114116426*, and *GRP*) were previously identified as DEGs (Table 3). In addition to the previously discussed genes associated with resistance to gastrointestinal nematodes (such as *GAST* and *IL13*), genes involved in adaptation (such as *ABCC11* [76] and *GNGT1* [77]) were detected.

**Table 4:** Mean counts per million (CPM) of genes exclusively expressed in the abomasum of Morada Nova sheep resistant or susceptible to *H. contortus*

Gene ID	CPM in resistant	CPM in susceptible	Gene name	Expressed
*CCDC85B*^a^	4.06	0.00	Coiled-coil domain containing 85B	Resistant
*LOC121819799*^a^	0.82	0.00	ncRNA	Resistant
*GAST*^a^	87.29	0.00	Gastrin	Resistant
*ABCC11*	0.53	0.00	ATP binding cassette subfamily C member 11	Resistant
*LOC101106468*	0.74	0.00	Transcription initiation factor TFIID subunit 9-like	Resistant
*LOC121818066*	1.16	0.00	ncRNA	Resistant
*LOC101104728*^a^	0.00	2.67	Antimicrobial peptide NK-lysin	Susceptible
*GNGT1*	0.00	0.75	G protein subunit gamma transducin 1	Susceptible
*LOC114116426*^a^	0.00	1.31	Protein C1orf43 homolog	Susceptible
*GRP*^a^	0.00	4.41	Gastrin releasing peptide	Susceptible

^a^ Detected in edgeR and/or DESeq2 analyses

From a total of 76,900 transcripts, 478 differentially expressed transcripts (DET) from 435 genes (including *LOC101116991*, *LOC114116426*, *LOC101107420*, and *IL13* DEGs) were detected using DESeq2 (Additional file 1: Table S3), whereas ballgown (Additional file 1: Table S4 and Additional file 4: Fig. S2) detected only three transcripts (*TECPR1*, *LOC114111361*, and *LOC121818463* genes).

### Variant calling and annotation of RNA-seq data

A total of 884,918 variants were detected in sheep abomasum RNA-seq data. After filtering, 51,434 autosomal SNPs remained and were associated with 264,604 effects, of which 0.04% had high impact and 24.8% had missense effects. Case–control selection of RNA-seq genotypes between resistant and susceptible sheep revealed 16 variants (Additional file 1: Table S5) in three genes: *TRAPPC6B* (Chr18), *MGRN1* (Chr24), and *SPCS3* (Chr26). Among these, *MGRN1* has been associated with resistance to gastrointestinal nematodes in a previous study [78].

### Genome-wide association study

After quality control filtering of the genotype data, 45,070 SNPs from 44 lambs were used in the snpBLUP GWAS. The observed SNP average heterozygosity was 0.38, and the diagonal mean value of the genomic relationship matrix (GRM) was 0.95, indicating no inbreeding in the population. This was also confirmed by the inbreeding coefficient estimation at 0 using the A matrix of the pedigree data. The animals used in this study were raised in the state of São Paulo, which holds 10.4% of the Morada Nova population registered in Brazil [79]. However, high levels of inbreeding have been reported in animals from northwestern Brazil [80]. Production conditions under extensive or semi-intensive systems and animal selection practices vary significantly between Brazilian states; consequently, the genetic background of the breed differs as well [81]. Nonetheless, Morada Nova animals exhibit sufficient genetic variation to be used in breeding programs [80].

The opposing homozygous matrix identified two discordant sire-offspring pairs that needed to be reassigned. In the final data of the 44 lambs, 11 were offspring of sire 1 (with a 28.1 × variation in EPG means between resistant and susceptible half-siblings), seven of sire 2 (7.5 × variation in EPG means), 13 of sire 3 (19.9 × variation in EPG means), and 13 of sire 4 (30.8 × variation in EPG means).

The h^2^ of EPG was estimated at 0.12 using the GRM and at 0.02 using the pedigree, noting the limited number of animals used to estimate h^2^, which also impaired the confidence interval calculation. The reported heritability estimates for EPG vary significantly in the range 0.01–0.65, as it is a complex trait that is easily affected by breed, age, parasite, climate, natural versus artificial parasite infection, and other experimental conditions [4]. Further studies with larger numbers of individuals are required to address the reliability of selecting Morada Nova individuals that are resistant to *H. contortus* based solely on EPG. However, even if not used for animal selection, the genomic estimated breeding value (GEBV) for EPG is considered a good criterion for identifying animals for target-selective anthelmintic treatment [82], which can contribute to the control of gastrointestinal nematodes in flocks.

**Fig. 1:**
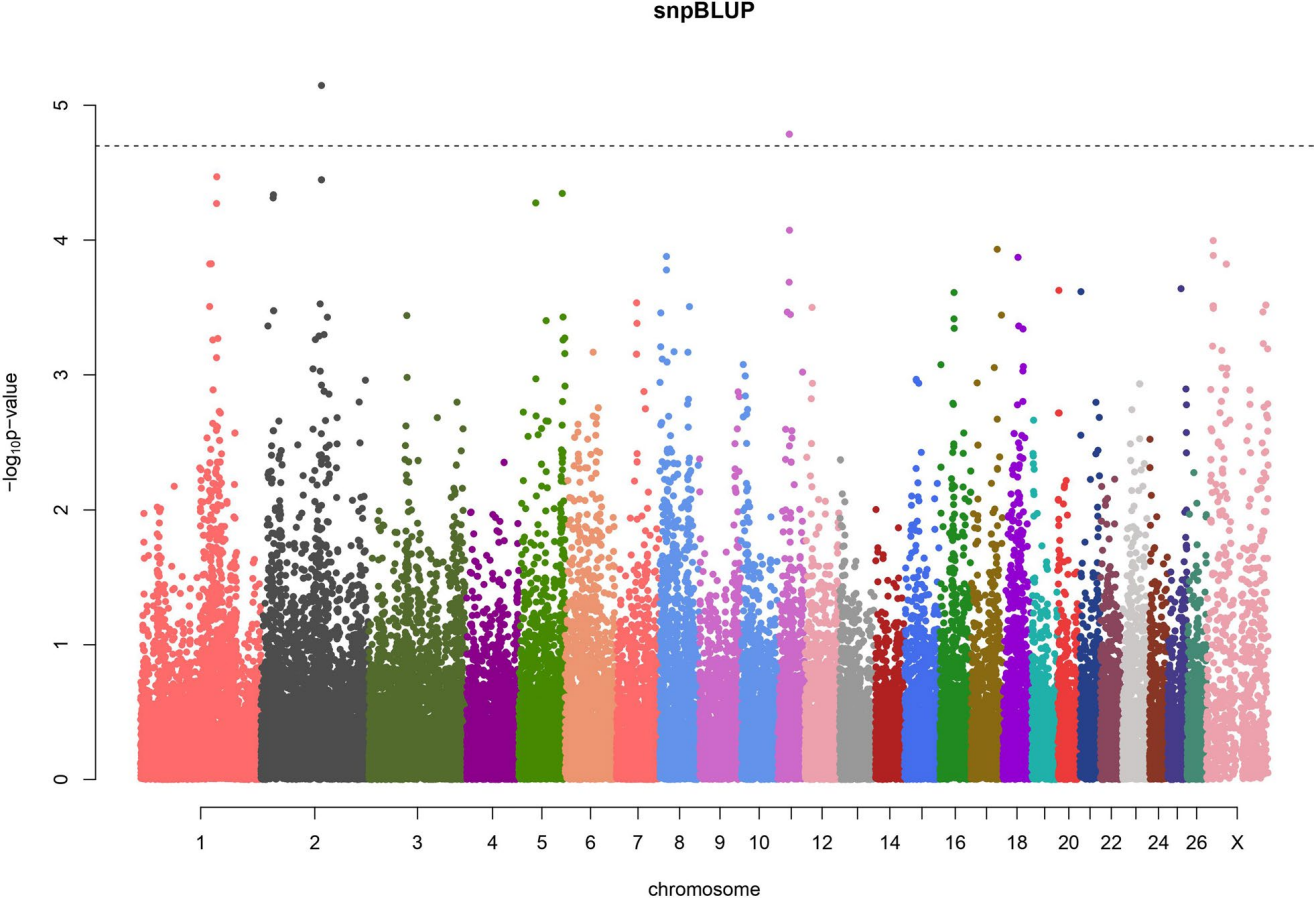
snpBLUP genome-wide association study for eggs per gram (EPG) in Morada Nova sheep. Dashed line: FDR < 0.05

The snpBLUP GWAS (Fig. 1) detected three significantly associated SNPs (FDR < 0.05): rs419988472 (intron variant in the *DHRS9* gene) at position Chr2:140323047, OAR11_27473453.1 (rs161041632—exon variant in the *MED11* gene) and s08310.1 (rs427555933 – intergenic variant) at positions Chr11:26501006 and Chr11:26595120, respectively, which overlapped in Fig. 1.

Within the ± 1 Mbp window of the significant SNPs, 171 annotated genes were detected (Additional file 1: Table S6). The candidates included genes with reported association with resistance to gastrointestinal nematodes, such as *NLGN2* in goats [83] and *CXCL16* and *CD68* in cattle [84]; specific response and resistance to *H. contortus*, such as *ALOX15* [73] and *LRP2* [85], to *T. circumcincta*, such as *RABEP1* [86], to the cestode *Echinococcus granulosus*, such as *TNFSF13* [87], and to the protozoan *Toxoplasma gondii* in mice, such as *NLRP1* [88]. In addition, candidate genes are related to macrophage regulation during helminth infection, such as *KDM6B* [89], response to *Ostertagia ostertagi* vaccination in cattle, such as *DHRS9* [90], and lipoxygenases (*ALOXE3*, *ALOX12*, *ALOX12B*, and *ALOX15*) associated with the inflammatory response in mice [91]. There are also genes related to body size and feed efficiency in sheep, such as *ALOX12* and *TP53* [92], and in cattle, such as *TP53* [93], *WSCD1* [94], and *TRNAC-GCA* [95], to adaptability, such as *SLC2A4* for hypoxia [96] and *CHD3* for disease resistance and climate adaptation in sheep [97], and *DVL2* [98] for thermal adaptation in cattle. Among the candidate genes involved in microbiota regulation, *ABCB11* (also known as *BSEP*) plays a role in the transport of bile salts, whose direct microbial activity shapes the gut microbiota [99], and *NLGN2* gene expression in horses is affected by anthelmintic treatment, likely through an effect on the microbiota [100].

### eQTL analysis

For gene expression, 48,682 significant (FDR < 0.05) eQTL consisting of 426 *cis*-eQTL (Additional file 2: Table S7) and 48,256 *trans*-eQTL (Additional file 2: Table S8) were detected, whereas transcript expression resulted in 9,999,256 eQTL: 2540 *cis*-eQTL (Additional file 2: Table S9) and 9,986,716 *trans*-eQTL (Additional file 2: Table S10). Among the significant eQTL, no DEG was regulated by significant variants. The lack of *cis*-eQTL for the significant variants suggests the absence of regulation among genes closely located on the same chromosome; however, the small sample size of this study has limited the power to identify smaller effects; a larger sample size generally results in gains for *cis*-eQTL mapping [101].

*Trans*-eQTL were detected for DETs (Table 5), including transcripts of functional candidate genes, such as *FGF14* associated with response to *H. contortus* in sheep [102] and *RORC* in goats [103]; *LRRC8B* associated with growth, body conformation, and weight [104]; *DTX3* [105] and *ZNF789* [97] associated with adaptation; and *RAPGEF2* [106] associated with microbiota.

**Table 5:** Significant eQTL between SNP variants and differentially expressed transcripts (DET) upregulated in *H. contortus*-resistant or *H. contortus*-susceptible Morada Nova sheep

SNP (gene—chromosome)	DET (gene—chromosome)	Upregulated
OAR11_27473453.1 and s08310.1 (*MED11* and intergenic—Chr11)	XM_042241996.1 (*TMEM187*—ChrX)	Susceptible
NC_056071.1:46893094_C/A (*TRAPPC6B*—Chr18)	XM_042256817.1 (*RORC*—Chr1), XM_042243310.1 (*LOC121818624*—Chr2), XM_027967527.2 (*DTX3*—Chr3), XM_042249264.1 (*AGAP3*—Chr4), XM_027970457.2 (*THG1L*—Chr5), XM_042252750.1 (*PTGR2*—Chr7), XM_042255599.1 (*CHRNE*—Chr11), XM_042230308.1 (*CDC123*—Chr13), XR_006056188.1 (*LOC101122718*—Chr14), XM_042234867.1 (*C17H22orf15*—Chr17), XM_042237011.1 (*BRD2*—Chr20), XR_003586743.2 (*KDSR*—Chr23), XM_042240704.1 and XM_042240703.1 (*TECPR1*—Chr24)	Resistant
	XM_015092088.3 (*LRRC8B*—Chr1), XM_042251691.1 (*HGFAC*—Chr6), XM_015097958.3 (*FGF14*—Chr10), XM_027977036.2 (*FAM110A*—Chr13), XM_042234479.1 and XM_042234478.1 (*EP400*—Chr17), XM_004021043.5 (*ZNF789*—Chr24), XR_003587432.2 (*LOC114111361*—ChrX), XR_006058459.1 (*LOC121818463*—unknown)	Susceptible
NC_056077.1:4352395_C/A and NC_056077.1:4352403_C/T (*MGRN1*—Chr24)	XM_042234385.1 (*RAPGEF2*—Chr17)	Resistant
NC_056079.1:6981437_A/G (*SPCS3*—Chr26)	XM_015097958.3 (*FGF14*—Chr10)	Susceptible

### Unmapped read analysis (RNA-seq from *H. contortus*)

Between 0.4% and 1.0% of the total abomasum RNA-seq data reads were mapped to *H. contortus*, resulting in 0.1–0.4 M. Most of the 19,776 annotated genes had zero counts, and only 40 genes (0.2%) were retained for further analyses.

The most highly expressed gene detected in all the samples was HCON_00026760, a non-annotated pseudogene. No DEG using DESeq2 (Additional file 1: Table S11), edgeR (Additional file 1: Table S12), or t-test, and no DET using DESeq2 (Additional file 1: Table S13) or ballgown (Additional file 1: Table S14) were detected between the reads of *H. contortus* retrieved from resistant and susceptible Morada Nova sheep.

Of the 40 genes, nine were exclusively expressed (Additional file 1: Table S15) in *H. contortus* collected from resistant Morada Nova sheep; however, only in one sample each, whereas 26 genes were expressed exclusively in *H. contortus* recovered from susceptible sheep (Additional file 1: Table S15), but only four in at least two samples: HCON_00174250 (Epsin-1), HCON_00667215 (Cytochrome c oxidase subunit 1), HCON_00667245 (NADH:ubiquinone reductase [H( +)-translocating]), and HCON_00667255 (NADH dehydrogenase subunit 6). Epsin-1 is an ENTH-domain protein involved in endocytosis and lysosomal protein trafficking, and its silencing in *Heterodera avenae,* a cereal cyst nematode, results in a 71% reduction in females and eggs [107]. Cytochrome c oxidase, NADH:ubiquinone reductase, and NADH dehydrogenase are mitochondrial genes, which are considered potential targets for anthelmintic treatments because of the unique energy-transducing and anaerobic systems developed by nematode parasites in their adaptation to the low oxygen concentration of the mammalian host gastrointestinal tract [108, 109]. Despite their expression in only one sample each, three genes related to collagen and cuticle composition were expressed exclusively in *H. contortus* recovered from susceptible sheep (Additional file 1: Table S15). The nematode cuticle interacts with the host immune system and its major protein is collagen [110]. Genes associated with collagen and cuticle development are upregulated in the transition from L_3_ to L_4_ in *H. contortus* [111].

The potential roles of epsin-1, mitochondrial, collagen-, and cuticle-related genes in the establishment and maintenance of infection and parasite fitness in sheep hosts should be further investigated in future studies and considered as potential targets for the development of new therapeutics against *H. contortus*.

### Gene co-expression network analysis

Early establishment of the gut microbiome is crucial for immune system development and maintenance of a healthy gut, including barrier function and mucosal immunity [112]. Moreover, the nematodes modulate their microbiome to provide an adequate environment for their survival [113]. The results obtained here from the microbiome may reflect the identification of taxa consistently associated with infection and resistance to *H. contortus* and must be interpreted carefully, as significant variations in bacterial populations were detected between trials in 2 years, consecutively, after immunization against and infection by *T. circumcincta* [114].

**Fig. 2:**
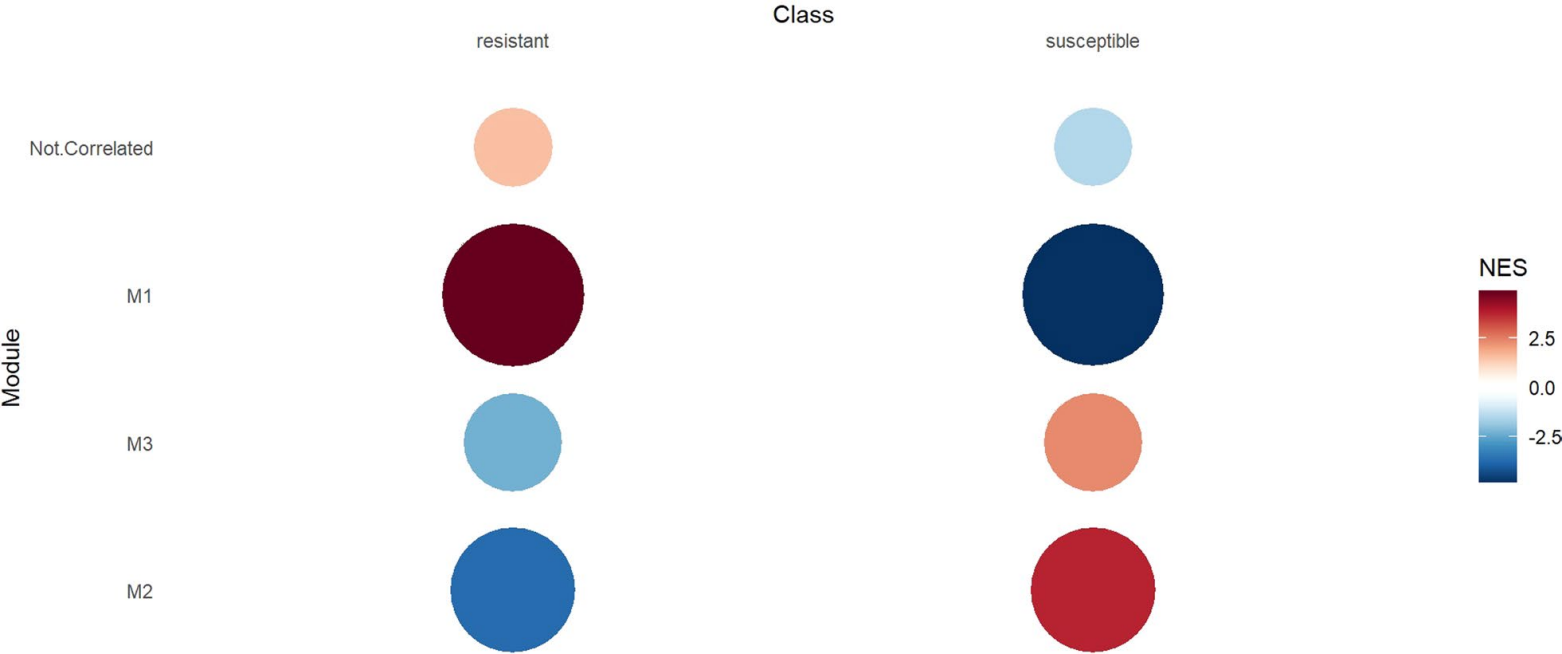
Co-expression network analysis of sheep RNA-seq genes and amplicon sequence variants (ASV). The figure displays the normalized enrichment score (NES) of modules (red represents higher activity and blue represents lower activity), with circle size and color intensity proportional to the NES values

Co-expression network analysis detected four functional modules based on the sheep RNA-seq gene data and ASVs (Fig. 2). Among these, three modules (Fig. 2 and Table 6) exhibited a significant association with resistance to *H. contortus*, whereas one module was not associated with the trait. Notably, the most active module, referred to as M1, encompassed 302 correlated features (77 ASVs) and displayed a positive normalized enrichment score (NES) of 4.87 in the resistant group. In contrast, M2, consisting of 284 features (117 ASVs), exhibited a negative NES of − 3.75 in the resistant group, and M3, containing 240 features (36 ASVs), also exhibited a negative NES of − 2.32 in the resistant group.

**Table 6:** Significant functional co-expression modules of sheep RNA-seq genes and amplicon sequence variants (ASV)

Module	Number of features	Hubs
M1	Genes = 225; archaea feces = 3; archaea rumen = 5; bacteria feces = 27; bacteria rumen = 42	*LOC114116187* (*CALHM6*), *CD8B*, *LOC114109611* (*UBD*), *CD8A*, *LOC101122689* (*TRGC1*)
M2	Genes = 167; archaea rumen = 9; bacteria feces = 54; bacteria rumen = 54	ASV_Bac_1273_R, ASV_Bac_422_S, *CCDC150*, ASV_Bac_396_R, *HKDC1*
M3	Genes = 204; archaea feces = 1; archaea rumen = 4; bacteria feces = 16; bacteria rumen = 15	*SLC26A7*, *HRH2*, *TNNI3*, *KCNJ16*, *PPP1R1A*

Hub genes/genera representative of each of the three modules associated with *H. contortus* resistance were also identified (Table 6 and Additional file 1: Tables S16 and S17). In the case of M1, the hub features were genes associated with the immune system, such as *CALHM6*, *CD8A*, *CD8B*, and *TRGC1*. *CD8A* and *CD8B* encode subunits of the CD8 protein that occurs on the surface of cytotoxic T cells. A DNA vaccine conferring partial protection against *H. contortus* infection in goats resulted in increased CD8 + T lymphocyte production and reduced EPG and worm burden in the abomasum [115]. *TRGC1* encodes the gamma-constant region of the T cell receptor surface, which recognizes and binds to specific antigens and plays a multifaceted role in tissue homeostasis, autoimmunity, pro- and antitumor activity, and innate and adaptive immune responses [116, 117]. However, no relationship between *TRGC1* and nematode infections has been previously described.

The M2 module had three ASVs as hub features: ASV_Bac_1273_R, classified as belonging to the *Christensenellaceae R-7 group* genus; ASV_Bac_396_R, classified as the *Kiritimatiellae* class (both found in the rumen); and ASV_Bac_422_S, classified as the *Bacteroides* genus, from the fecal samples. In addition, the *HKDC1* hub gene from M2, which exhibited reduced activity in resistant animals, is associated with glucose use and homeostasis [118].

*SLC26A7* and *KCNJ16* are hub genes in the M3 module that exhibited lower activity in resistant animals. These genes are related to homeostasis and pH balance [119] and, in addition to *HRH2*, they impair gastric acid secretion [120, 121]. Previous studies have hypothesized that gastric acid protects against nematode infections, as shown by reduced gastric acid secretion and predisposition to infection by a variety of nematodes, including *Ostertagia* spp. and *T. colubriformis* in sheep [122, 123]. *H. contortus* infection causes a significant increase in the abomasal pH during the early and late stages of infection in goats [124]. Our findings present a correlation between gastric acid secretion and susceptibility to *H. contortus*. Therefore, compared to resistant animals, susceptible animals are less able to respond to *H. contortus* infection through gastric acid secretion, which, in turn, results in higher infection rates.

**Fig. 3:**
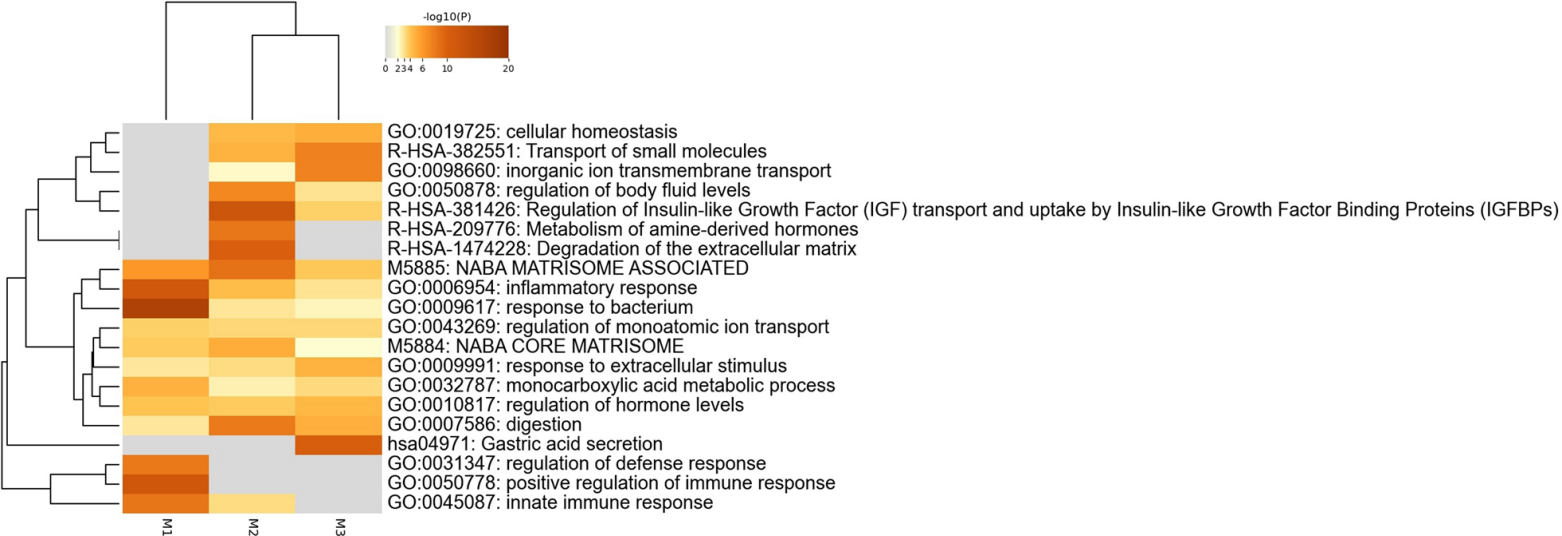
Metascape enrichment analysis of statistically enriched ontology terms of hub genes/genera from three functional co-expression modules of sheep RNA-seq genes and amplicon sequence variants (ASV). Heatmap of enriched terms across input gene lists, colored by *p*-values

The annotated genes were subjected to pathway analysis to capture biological information using enriched terms (Fig. 3 and Additional file 1: Table S18). For genes in M1, the top pathways and processes included responses to bacteria and positive regulation of immune and inflammatory responses, which are traits intrinsically related to parasite resistance. Nematodes have evolved immunomodulatory mechanisms to suppress host immune responses and promote infection [125]. Knowledge of the mechanisms and target molecules involved in the inflammatory response may provide an effective means of nematode parasite control [126]. For genes in M2, the enriched terms were related to the regulation of insulin-like growth factor transport and uptake by insulin-like growth factor binding proteins, post-translational protein phosphorylation, and degradation of the extracellular matrix. In addition, for genes in M3, gastric acid secretion was highlighted.

Enrichment of the genera classified as *Prevotella*, *Treponema*, *Christensenellaceae* R-7 group, and *Methanobrevibacter* was observed in the M1 and M2 modules. Four ASVs were observed in both the rumen and feces in M1: ASV_98 and ASV_23, classified as the *p-251-o5* and [*Eubacterium*] *coprostanoligenes* group bacterial families, and ASV_13 (*Methanobrevibacter* genus) and ASV_6 (*Candidatus Methanomethylophilus*), which are two archaea. For M2, five archaeal ASVs, *i.e.*, ASV_3, ASV_4, ASV_8, ASV_31, and ASV_57, which are classified as *Methanobrevibacter*, were enriched. Among the ASVs, the *p-251-o5* family identified in M1 has been described in the fecal microbiota of pigs [127] and in the rumen of ewes and cows [128, 129]. In addition, the *[Eubacterium] coprostanoligenes* group, an anaerobic bacterium potentially impacting host lipid metabolism [130], was less abundant in Tibetan sheep infected with gastrointestinal nematodes [131], supporting our results and suggesting that it may be a promising target for *H. contortus* resistance. *Christensenellaceae R-7*, a hub feature in M2, is associated with amino acid and lipid metabolism and human metabolic health in different disease contexts, including obesity and inflammatory bowel disease [132]. The relative abundance of *Christensenellaceae* decreases in goats infected with *H. contortus* [124]. The *Bacteroides* genus, an ASV from feces in the M2 module, is considered beneficial to hosts as it prevents potential pathogens from colonizing the gut and processes complex molecules into simpler molecules [133].

The hub archaeal ASV, classified as *Methanobrevibacter* genus in the M2 module, plays a notable role in the gut microbial ecosystem [134] by contributing to the efficient digestion of polysaccharides by consuming the end products of bacterial fermentation [135]. It is considered a potential therapeutic target for managing gastrointestinal disorders [136]. Anthelmintic treatment of adult ewes significantly affects the archaeal community, resulting in increased relative abundance of different *Methanobacteria* [137]. An increase in *Methanobrevibacter* has been observed during chronic *Trichuris trichiura* infections in humans [138]. It was hypothesized that *H. contortus* infection could increase mucus secretion [139], which would provide energy for adapted microorganisms, including the mucus colonizer *Methanobrevibacter* [138]. Thus, the potential role of *Methanobacteria* in controlling *H. contortus* infections is of interest.

### Transcript co-expression network analysis

Co-expression network analysis of RNA-seq transcript data identified 26 functional co-expression modules (Additional file 5: Figure S3 and Additional file 1: Table S19) and hub features (Additional file 1: Table S20). The top modules and hub features (Table 7) were M2 (294 features with 55 ASVs, NES = − 3.85), M25 (123 features with seven ASVs, NES = − 3.29), M6 (263 features with 31 ASVs, NES = 3.66), and M10 (231 features with eight ASVs, NES = 3.4).

**Table 7:** Top significantly functional co-expression modules of sheep RNA-seq transcripts and amplicon sequence variants (ASV)

Module	Number of features	Hubs
M2	Isoforms = 239; archaea feces = 2; archaea rumen = 3; bacteria feces = 24; bacteria rumen = 26	ASV_Bac_396_R, ASV_Bac_250_R, XM_042238037.1 (*SYTL2*), ASV_Bac_1273_R, ASV_Bac_422_S
M6	Isoforms = 232; archaea feces = 3; archaea rumen = 4; bacteria feces = 11; bacteria rumen = 13	ASV_Bac_1254_R, ASV_Bac_433_R, ASV_Bac_115_R, ASV_Bac_269_R, ASV_Bac_218_S
M10	Isoforms = 223; archaea rumen = 1; bacteria rumen = 7	XM_027958292.2 (*SFMBT1*), XM_042256129.1 (*MAPT*), XM_042230866.1 (*ZNF570*), ASV_Bac_239_R, XM_027964183.2 (*SEMA4D*)
M25	Isoforms = 116; bacteria feces = 4; bacteria rumen = 3	XM_042250691.1 (*PWWP2A*), XM_027978299.2 (*COQ8B*), XM_012180311.3 (*PTPN13*), XM_042247173.1 (*RIC8B*), XM_042237043.1 (*SSR1*)

The M2 and M25 modules of the transcript networks were less active in resistant animals. Regarding M2, several ASVs were identified as hubs, including the same ASVs found in the gene level M2 module. ASV_Bac_250_R, classified as *Verrucomicrobiota* and identified as a hub for M2, is associated with mucin degradation, glucose homeostasis, and immunity regulation [140]. Previous studies have described a symbiotic association between *Verrucomicrobia* and soil nematodes [141], and the abundance of the *Verrucomicrobiota* phylum was decreased in the abomasum of lambs infected with *H. contortus* [142], suggesting potential mechanisms for therapeutic interventions.

For M6, five ASVs were identified as hubs: ASV_Bac_1254_R (*Prevotella*), ASV_Bac_433_R (*Ruminococcus gauvreauii* group genus), ASV_Bac_115_R (*Prevotellaceae NK3B31* group genus), ASV_Bac_269_R (*Prevotella*), and ASV_Bac_218_S (*Lachnospiraceae*). In addition, previous studies have shown an increase in *Prevotella* abundance in the ruminal, fecal, and abomasal microbiota of sheep and goats infected with *H. contortus* [15, 143]. In the present study, a decrease in *Prevotella* abundance associated with *H. contortus* susceptibility was observed, which is consistent with more recent findings after *H. contortus* infection [124, 142] and other nematode infections in humans [144] and mice [145]. In addition, as the *Prevotella* genus specializes in complex polysaccharide degradation, such as starch and cellulose, and contributes to the metabolism of dietary fiber [146], its reduction may affect feed digestion, resulting in low weight gain in infected animals [145] and, consequently, lower resilience to parasites.

**Fig. 4:**
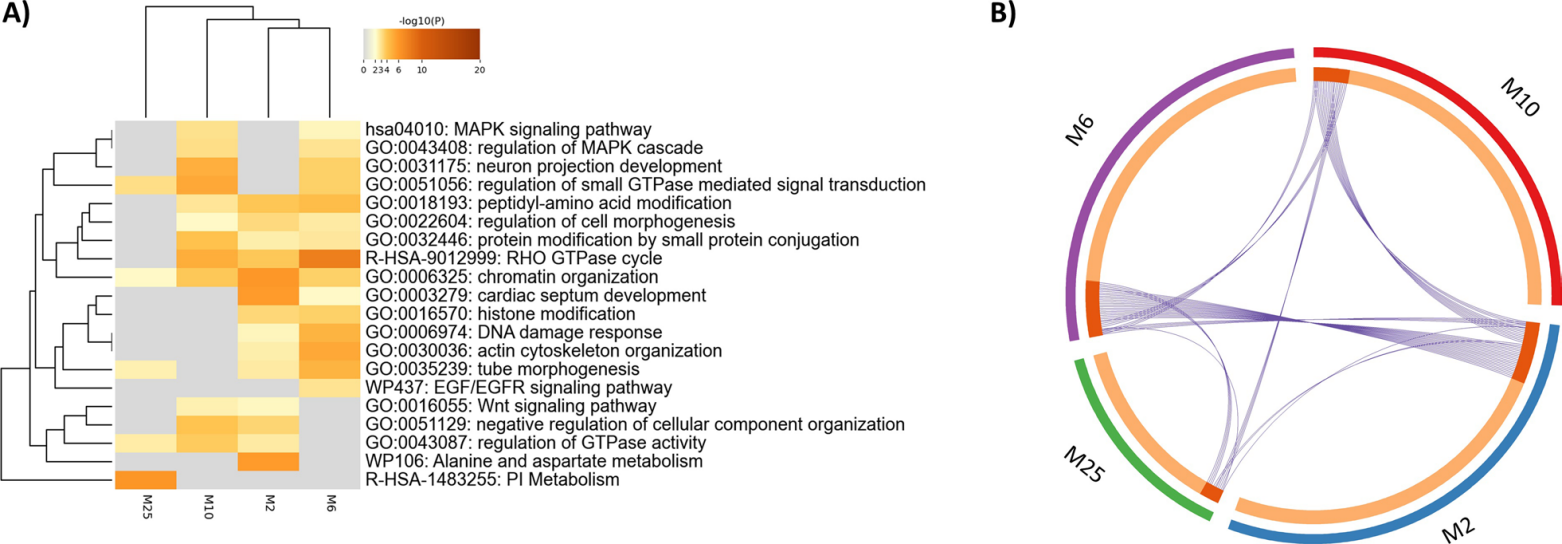
**A** Metascape enrichment analysis of statistically enriched ontology terms. Heatmap of enriched terms across input gene lists, colored by *p*-values. **B** Overlap among gene lists: identical genes are linked with purple curves, genes that hit multiple lists are colored with dark orange, and genes unique to a list are colored with light orange

Regarding pathway-enriched terms (Fig. 4 and Additional file 1: Table S21), the genes associated with each transcript were enriched in the MAPK, Wnt, and EGF/EGFR signaling pathways, morphogenesis, and cellular processes, including the regulation of cell morphogenesis, chromatin organization, and actin cytoskeleton organization. Additionally, transcripts representing the same genes (*i.e.*, *ABO*, *ASAP2*, *ADGRF5*, *ARHGAP12*, *CAPN15*, and *FN1*) were identified in various modules.

None of the *H. contortus* genes or transcripts was significantly correlated with the identified modules.

## Conclusions

Gene and transcript expression data and genomic markers from Morada Nova sheep were investigated and associated using eQTL to assess their roles in the phenotype of *H. contortus* resistance. Some candidate genes were related to immune response and parasite resistance (*ALOX12*, *ALOX12B*, *ALOX15*, *CD68*, *CXCL16*, *DHRS9*, *DNAH2*, *FGF14*, *GAST*, *GNLY*, *GP1BA*, *IL13*, *KDM6B*, *LRP2*, *MGRN1*, *NLRP1*, *RABEP1*, *RORC*, and *TNFSF13*), growth and weight (*ENO3*, *TP53*, *LRRC8B*, *TRNAC-GCA*, and *WSCD1*), environmental adaptation (*CHD3*, *DTX3*, *DVL2*, *GNGT1*, *SLC2A4*, *TRNAG-CCC*, and *ZNF789*), and microbiota regulation (*ABCB11*, *NLGN2*, and *RAPGEF2*). Through eQTL mapping, SNP variants were predicted to regulate the differential expression of transcripts, including candidate genes (*DTX3*, *FGF14*, *LRRC8B*, *RORC*, and *SNF789*). These genes represent potential molecular markers for the detection and selection of *H. contortus-*resistant animals. Transcriptomic and genomic data from hosts, expression data from *H. contortus*, and ASVs from the microbiota of feces and rumen were integrated to detect the biological functions and pathways resulting in resistance. The genes (mitochondrial, collagen-, and cuticle-related genes) expressed in *H. contortus* may modulate evasion from the host immune system, facilitating the establishment of infection. Furthermore, the sheep genes (*CD8A*, *CD8B*, *HKDC1*, *KCNJ16*, *SLC26A7*, and *TRGC1*) and biological pathways (positive regulation of the immune system, response to bacteria, inflammatory response, gastric acid secretion, and mucus secretion) identified using this multi-omics approach are potential modulators of host immunity. Additionally, enriched microbiota (*Bacteroides*, *Christensenellaceae R-7*, *[Eubacterium] coprostanoligenes* group, *Methanobrevibacter*, *Prevotellaceae*, and *Verrucomicrobiota*) may regulate host metabolism (homeostasis, glucose use, amino acid and lipid metabolism, and dietary fiber degradation), resulting in resistance to parasite infection. The parasitic genes, biological pathways, and microbiomes identified in this study are potential targets for the development of new therapeutics aimed at increasing host resistance and parasitic control in sheep flocks.

### Supplementary Information

**Additional file 1: Table S1. **edgeR analysis of differential gene expression in the abomasum between *H. contortus*-susceptible and *H. contortus*-resistant Morada Nova sheep. **Table S2. **DESeq2 analysis of differential gene expression in the abomasum between *H. contortus*-susceptible and *H. contortus*-resistant Morada Nova sheep. **Table S3.** DESeq2 analysis of differential transcript expression in the abomasum between *H. contortus*-susceptible and *H. contortus*-resistant Morada Nova sheep. **Table S4. **Ballgown analysis of differential transcript expression in the abomasum between *H. contortus*-susceptible and *H. contortus*-resistant Morada Nova sheep. **Table S5.** Annotation and impact of homozygous SNPs between resistant and susceptible Morada Nova sheep detected after case-control variant calling of RNA-seq data. **Table S6.** Genes located in the +/- 1 Mbp window of each significant SNP by GWAS for EPG in Morada Nova sheep.** Table S11. **DESeq2 analysis of differential gene expression in *H. contortus* recovered from resistant and susceptible Morada Nova sheep. **Table S12. **edgeR analysis of differential gene expression in *H. contortus* recovered from resistant and susceptible Morada Nova sheep. **Table S13. **DESeq2 analysis of differential transcript expression in *H. contortus* recovered from resistant and susceptible Morada Nova sheep. **Table S14. **Ballgown analysis of differential transcript expression in *H. contortus* recovered from resistant and susceptible Morada Nova sheep. **Table S15.** Genes expressed in *H. contortus* recovered from resistant and susceptible Morada Nova sheep. **Table S16.** Gene-level features and ASV modules associated with resistance to *H. contortus*. **Table S17.** ASV taxonomy of gene co-expression modules from sheep feces and rumen content associated with resistance to *H. contortus*. **Table S18.** Gene-level relevant pathways and ASV modules in *H. contortus*-resistant and *H. contortus*-susceptible Morada Nova sheep. **Table S19.** Transcript-level features and ASV modules associated with resistance to *H. contortus*. **Table S20.** Transcript functional co-expression modules and ASVs associated with resistance to *H. contortus*. **Table S21.** Transcript-level relevant pathways and ASV modules in *H. contortus*-resistant and *H. contortus*-susceptible Morada Nova sheep.

**Additional file 2: Table S7.** *Cis*-eQTL for differentially expressed genes between resistant and susceptible Morada Nova sheep. **Table S8.** *Trans*-eQTL for differentially expressed genes between resistant and susceptible Morada Nova sheep. **Table S9. ***Cis*-eQTL for differentially expressed transcripts between resistant and susceptible Morada Nova sheep. **Table S10. ***Trans*-eQTL for differentially expressed transcripts between resistant and susceptible Morada Nova sheep.

**Additional file 3: Figure S1.** Differential gene expression and count in abomasum of *H. contortus*-resistant and *H. contortus*-susceptible Morada Nova sheep for 11 genes detected using edgeR.

**Additional file 4: Figure S2. **Differential transcript expression and count in abomasum of *H. contortus*-resistant and *H. contortus*-susceptible Morada Nova sheep for three transcripts detected using ballgown.

**Additional file 5: Figure S3.** Co-expression analysis of sheep RNA-seq transcripts and amplicon sequence variants (ASV). The figure displays the normalized enrichment score (NES) (red represents higher activity and blue represents lower activity), with the circle size and color intensity proportional to the NES values.

## Data Availability

The datasets generated and/or analyzed during the current study are available in the European Nucleotide Archive repository, under the project PRJEB67593, https://www.ebi.ac.uk/ena/browser/view/PRJEB67593.

## Abbreviations

A matrix: Additive genetic relationship matrix
ASV: Amplicon sequence variant
DEG: Differentially expressed gene
DET: Differentially expressed transcript
EPG: Eggs per gram of feces
eQTL: Expression quantitative trait loci
FC: Fold change
FDR: False discovery rate
GEBV: Genomic estimated breeding value
GRM: Genomic relationship matrix
GWAS: Genome-wide association study
h^2^: Heritability
L_3_: Third-stage larvae
L_4_: Fourth-stage larvae
NES: Normalized enrichment score
PCR: Polymerase chain reaction
PE: Paired-end
RNA-seq: Transcriptome sequencing
SNP: Single nucleotide polymorphism
