# Supplementary figures and images for: Multi-omics data elucidate parasite-host-microbiota interactions and resistance to Haemonchus contortus in sheep
Source: Parasit Vectors. 2024 Mar 1;17:102. doi: 10.1186/s13071-024-06205-9 (PMC10908167; doi:10.1186/s13071-024-06205-9)

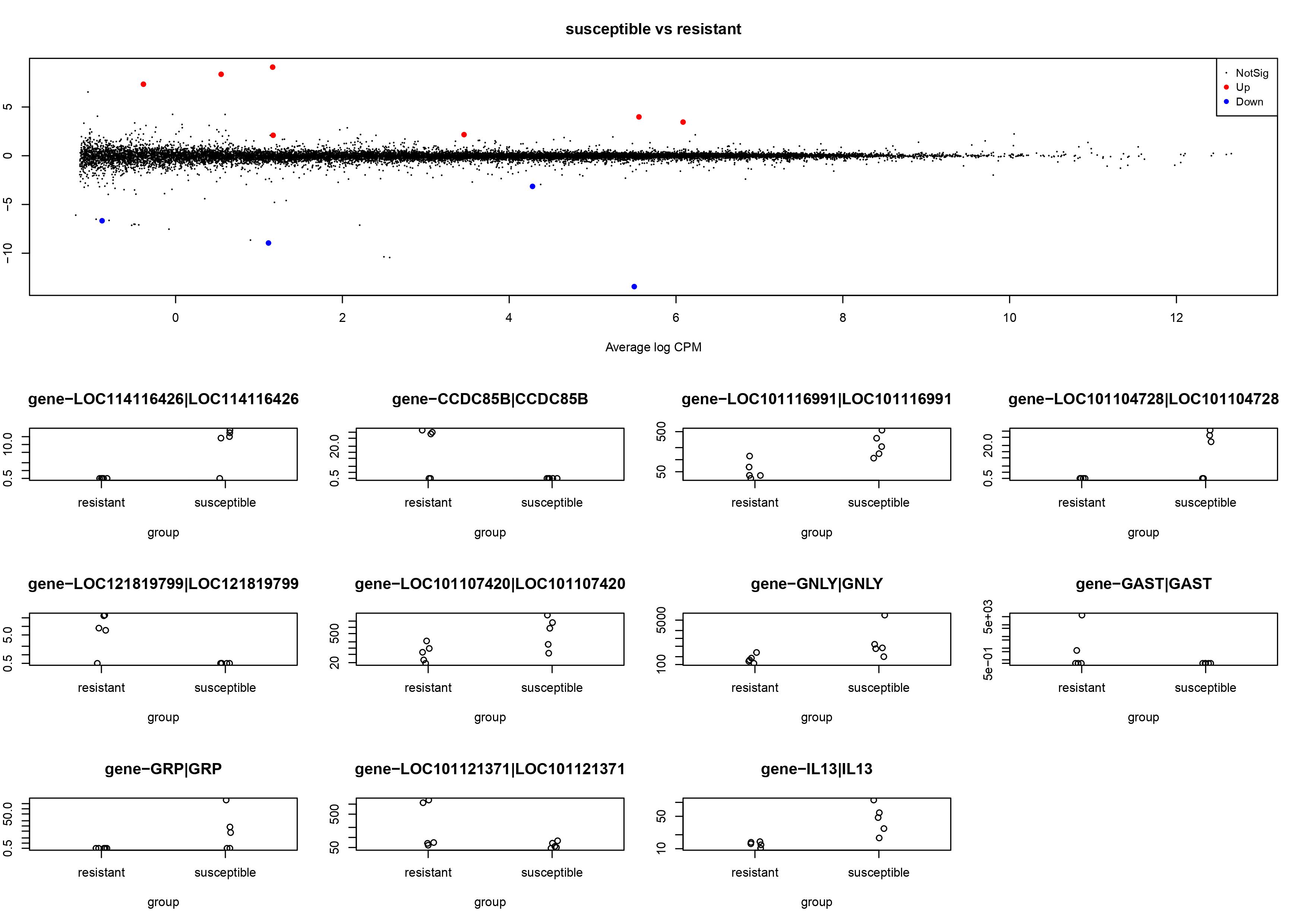

Supplement: Supplementary file 3 — Additional file 3: Figure S1. Differential gene expression and count in abomasum of H. contortus-resistant and H. contortus-susceptible Morada Nova sheep for 11 genes detected using edgeR. [file 13071_2024_6205_MOESM3_ESM.jpg]

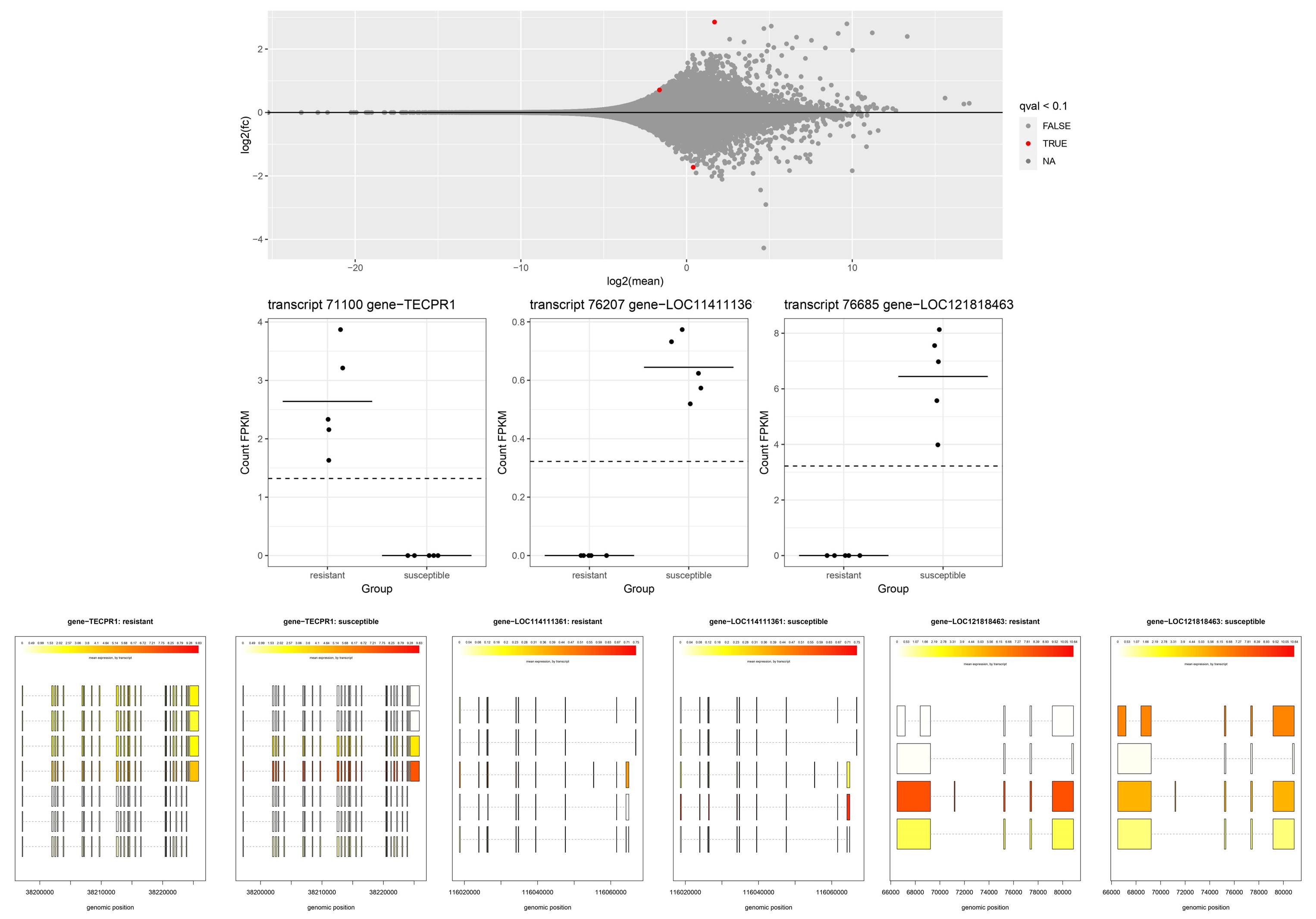

Supplement: Supplementary file 4 — Additional file 4: Figure S2. Differential transcript expression and count in abomasum of H. contortus-resistant and H. contortus-susceptible Morada Nova sheep for three transcripts detected using ballgown. [file 13071_2024_6205_MOESM4_ESM.jpg]

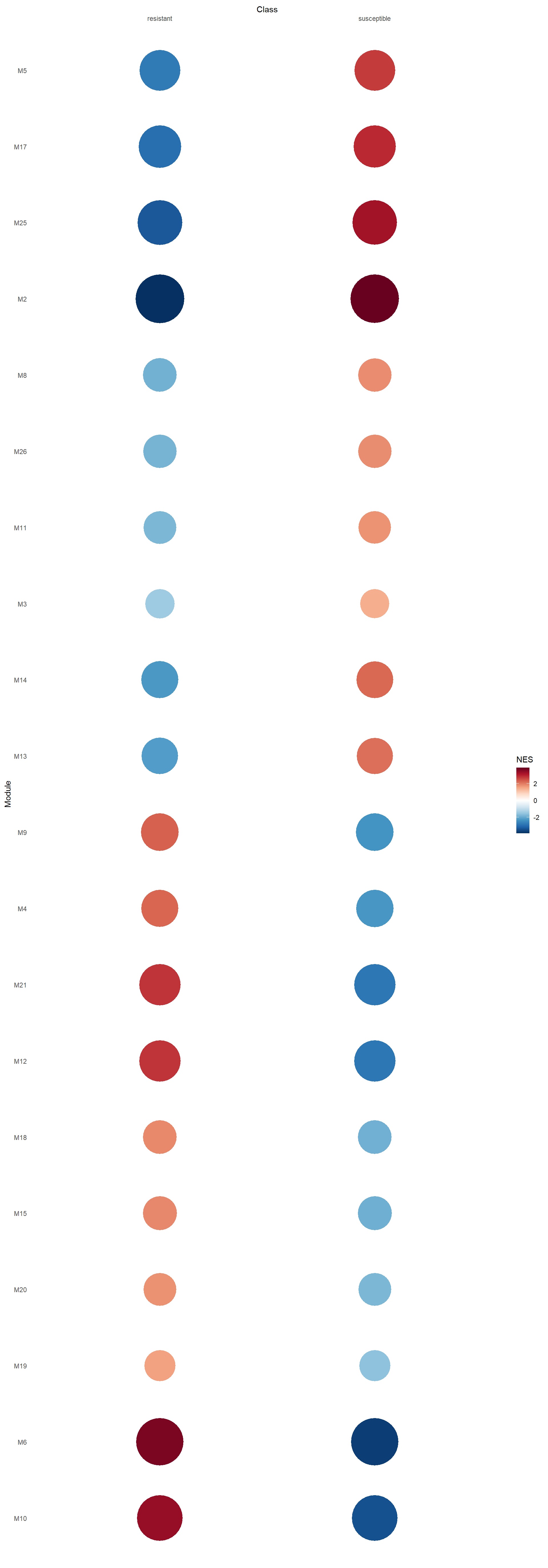

Supplement: Supplementary file 5 — Additional file 5: Figure S3. Co-expression analysis of sheep RNA-seq transcripts and amplicon sequence variants (ASV). The figure displays the normalized enrichment score (NES) (red represents higher activity and blue represents lower activity), with the circle size and color intensity proportional to the NES values. [file 13071_2024_6205_MOESM5_ESM.jpg]
